# Ethical and Practical Dimensions of Artificial Intelligence (AI) in Healthcare: A Comprehensive Study of Professional Perceptions

**DOI:** 10.7759/cureus.78416

**Published:** 2025-02-03

**Authors:** Esteban Zavaleta-Monestel, Adriana Anchía-Alfaro, Carolina Rojas-Chinchilla, Diego Fabian Quesada-Loria, Sebastián Arguedas-Chacón

**Affiliations:** 1 Pharmacy, Hospital Clínica Bíblica, San José, CRI; 2 Research, Hospital Clínica Bíblica, San José, CRI

**Keywords:** artificial intelligence in healthcare, bioethics, healthcare professionals, health policy and regulation, medical data privacy

## Abstract

Introduction

Artificial intelligence (AI) transforms medicine by enhancing diagnoses, treatments, resource management, and personalized treatment plans. However, it poses ethical and legal challenges, such as data privacy and equitable access to its benefits. This study seeks to understand healthcare professionals' perceptions of AI regulation in a Costa Rican hospital and analyze the alignment of Latin American regulations with local realities.

Methods

The research is qualitative, descriptive, and cross-sectional, focusing on AI guidelines and laws in health at both international and national levels. The sample includes healthcare professionals from a private hospital in Costa Rica. Two instruments were used: a documentary review and an online survey. Data analysis was performed using descriptive and correlational statistics with RStudio (R Foundation for Statistical Computing, Vienna, Austria (https://www.R-project.org/)).

Results

Eighty healthcare professionals participated in the study. Findings revealed that most exhibited moderate familiarity with AI while underscoring the critical need for robust governance frameworks to navigate the ethical and regulatory complexities surrounding its implementation. Notably, no significant correlation emerged between AI familiarity and demographic factors. Limitations of this study include its focus on a single hospital and the heterogeneous regulatory landscape across Latin American countries.

Conclusions

The study reveals that the integration of AI in healthcare is promising but complex, requiring a multidimensional approach that includes technical, ethical, and social aspects. Healthcare professionals in Costa Rica show a favorable disposition towards AI, recognizing its potential to improve healthcare, although they also highlight concerns about data privacy, security, and ethics.

## Introduction

Artificial intelligence (AI) encompasses technologies that empower computers to emulate human intelligence, enabling them to analyze, learn, interpret, and execute tasks. A comprehensive understanding of AI requires the exploration of diverse subfields within computer science, including machine learning, natural language processing, robotics, and computer vision [1]. 

AI has emerged as one of the most innovative medical technologies, offering significant advances that have transformed the way diseases are diagnosed and treated, healthcare resources are managed, and treatment plans are personalized. Its ability to analyze large volumes of data, identify complex patterns, and generate accurate predictions makes AI a powerful tool in healthcare [2]. 

AI has been successfully applied across various domains of modern medicine, including healthcare administration, medical image analysis, drug discovery, cancer research and treatment, and patient health monitoring. A specialized subfield, "AI-based diagnostics," has emerged, leveraging AI to effectively rule out and differentiate between numerous potential diagnoses for individual patients [3]. However, these technological advancements also pose significant challenges in governance and bioethics that require urgent attention [2]. 

AI in medicine extends beyond technological innovation to encompass significant ethical and legal considerations. As AI tools are embedded within healthcare systems, fundamental questions arise concerning data privacy, liability in the event of errors, and equitable access to AI's benefits [4]. The reliance on AI for managing vast amounts of data heightens concerns regarding the protection of sensitive personal and medical information. Data breaches compromise patient privacy, erode public trust in healthcare systems, and may lead to severe legal consequences [2,5]. 

A critical issue involves legal liability when AI systems make errors that result in patient harm. Developing a well-defined legal framework that delineates responsibilities is essential. Without such a framework, the risks associated with AI in medicine could outweigh its benefits [3]. 

Equity in healthcare also presents a significant challenge. There is a risk that the benefits of AI will remain confined to specific groups, exacerbating existing disparities in healthcare access. Moreover, improperly developed AI algorithms can perpetuate or even amplify biases present in underlying data, making it crucial to ensure that AI applications are inclusive and equitable, serving diverse patient populations [5,6]. 

The laws and regulations governing AI in medicine vary significantly across countries, adding to the complexity of its implementation. The lack of harmonization in international regulations impedes the safe and ethical application of AI on a global scale [2,4]. 

The general objective of this study was to understand the perceptions and attitudes of healthcare professionals in a Costa Rican hospital regarding the regulation of AI in the health sector and to analyze the alignment of Latin American regulations with local realities. The specific objectives included evaluating current laws, regulations, and standards governing AI in medicine and health systems at the international level; identifying the main bioethical challenges associated with AI implementation in these systems; analyzing the perceptions of healthcare professionals in a private general hospital in Costa Rica regarding AI integration in the health sector; and providing recommendations for public policies and medical practices that ensure effective governance and a bioethical approach to AI use. 

Although AI technologies have already been implemented in various areas of medicine, significant legal and ethical challenges remain global. One primary concern is privacy, particularly the collection and handling of patients' personal information. Documented cases have highlighted a lack of accountability in instances where patients' information was stolen or shared without authorization [3]. Informed consent for data collection must be obtained comprehensively, detailing why, how, and when data are collected, especially regarding images of patients in vulnerable states [7]. 

Another significant concern is the potential for discrimination, which can perpetuate injustice and inequality, distort doctor-patient relationships, undermine patient autonomy and consent, and ultimately cause harm [8]. Biased AI algorithms may lead to unfair decisions, erroneous predictions, and discriminatory practices. Therefore, while AI offers opportunities to improve clinical care, mitigating its risks is essential [3]. 

Globally, each country establishes regulations for AI use in healthcare. However, there is a notable lack of consolidated international regulations providing a common framework for implementation and supervision. This absence of unified global regulations creates challenges for adopting and monitoring AI technologies, especially when used transnationally or in interconnected contexts. Even developed countries, such as the United States and several European Union members, remain in the preliminary stages of regulation, with many offering only proposals or recommendations instead of definitive legal frameworks [9,10]. 

In the Latin American context, Peru stands out as the only country to establish an official legal framework for AI use in medicine. This progress represents a significant step for the region, though further efforts are needed for practical application and oversight. Other Latin American countries included in this study, such as Argentina, Brazil, Mexico, Colombia, and Costa Rica, have developed well-structured drafts or bills addressing key aspects of AI regulation. These drafts demonstrate growing interest and commitment by governments to regulate these technologies ethically and effectively [11-20]. 

Globally, regulatory proposals emphasize critical principles such as privacy and the protection of personal data. They also focus on respecting, protecting, and promoting human rights in AI applications. Justice, non-discrimination, and equity are core principles addressed in most frameworks to ensure AI systems do not perpetuate bias or violate human dignity. Additionally, regulations highlight the importance of transparency, risk management, and continuous clinical validation to ensure AI technologies' safety and efficacy in healthcare [11-20]. 

This study investigated healthcare professionals' perceptions of AI regulation in a Costa Rican hospital and analyzed the alignment of Latin American regulations with local realities. By evaluating existing regulations and identifying associated bioethical challenges, this study aims to contribute to a governance framework that ensures the safe, ethical, and equitable implementation of AI in healthcare, enabling the effective navigation of the globalized regulatory landscape. 

## Materials and methods

Research design 

The research was qualitative, descriptive, and cross-sectional, focusing on AI guidelines and laws in health at both international and national levels. The study included healthcare professionals from Hospital Clínica Bíblica, San José, Costa Rica. Two instruments were used: a documentary review and an online survey (Appendices). The survey, created and validated by Pal, was originally conducted on health professionals in India [21].

Population and sample 

The study included health professionals employed at Hospital Clínica Bíblica in San José, Costa Rica, encompassing doctors, pharmacists, nurses, psychologists, and microbiologists. Surveys with unanswered or incomplete questionnaires were excluded from the analysis.

Variables 

The study analyzed existing and proposed guidelines and laws regulating the development and application of AI in healthcare and medicine in the European Union [9], the United Kingdom [11,22], the United States [10], Argentina [12], Brazil [13], Chile [14], Colombia [15], Costa Rica [16], Ecuador [17], Mexico [18], Panama [19], Peru [20], and Uruguay [23]. Several factors guided the selection of countries for this study. To ensure comprehensive regional coverage, the analysis included a representative selection of Latin American countries, focusing on the Latin American context and Costa Rica specifically. Additionally, the European Union, the United Kingdom, and the United States, recognized leaders in AI development and regulation, provide valuable insights from regions with established and evolving regulatory frameworks. This diverse selection allows for a nuanced analysis of different regulatory strategies and their potential implications.

Survey variables included age, gender, profession, years of experience, hospital department, knowledge and attitudes about AI in health, ethics, and privacy, existing legislation, and professional practice and ethics in the use of AI.

Measurement instruments, techniques, and procedures 

This study employed two primary instruments. First, a document review analyzed guidelines from international organizations such as the World Health Organization (WHO), the United Nations Educational, Scientific and Cultural Organization (UNESCO), and the United Nations (UN) [24-26], alongside national legislation from various countries, including Costa Rica. An analysis matrix was utilized to compare these regulations and assess their suitability for the healthcare sector.

Second, an online survey consisting of 21 questions was administered to healthcare professionals between September 20 and September 30, 2024, to assess their knowledge, attitudes, and concerns regarding AI in medicine. The survey, originally developed and validated by Pal [21], was modified to suit the local context and research objectives of this study. These modifications included adapting the questions to align with Costa Rican healthcare systems and AI regulations. To ensure the questionnaire was culturally and linguistically appropriate, it was translated into Spanish, the primary language spoken by the participants.

The validity and reliability of the modified questionnaire were reassessed to ensure its applicability to the study. Content validity was evaluated through expert review by a panel of five professionals with expertise in AI, bioethics, and healthcare. Their feedback was incorporated into the final version of the questionnaire.

The survey was distributed online via secure email links sent directly to healthcare professionals working at Hospital Clínica Bíblica in Costa Rica. Participation was voluntary, and anonymity was ensured to encourage honest responses.

Statistical analysis and data management 

A comprehensive analysis of the collected data was conducted, employing both descriptive and correlational statistical methods. Qualitative data were presented in the form of percentages. The chi-squared test was used to assess the statistical significance of differences between proportions. Data analysis was performed using RStudio (R Foundation for Statistical Computing, Vienna, Austria (https://www.R-project.org/)).

## Results

Table 1 presents a detailed comparative analysis of international guidelines and national legislation relevant to the health sector. This table serves as a foundation for understanding the alignment and divergences between global frameworks and country-specific regulations.

**Table 1:** Comparative analysis of international guidelines and national legislation in the health sector WHO: World Health Organization; UNESCO: United Nations Educational, Scientific and Cultural Organization; UN: United Nations; AI: artificial intelligence References: [24-26]

Institution	Principle	European Union	United Kingdom	Argentina	Brazil	Chile	Colombia	Costa Rica	Ecuador	Mexico	Panama	Peru	Uruguay	United States
WHO	Documentation and transparency	Covered	Covered	Covered	Covered	Covered	Covered	Covered	Covered	Not covered	Covered	Covered	Covered	Covered
	Risk management and lifecycle approach to AI systems development	Covered	Improvements proposed	Covered	Covered	Covered	Partially Covered	Covered	Covered	Not covered	Not covered	Not covered	Covered	Covered
	Proposed uses and analytical and clinical validation	Covered	Covered	Covered	Covered	Covered	Covered	Partially Covered	Covered	Not covered	Not covered	Not covered	Covered	Covered
	Quality of AI-related data	Covered	Covered	Covered	Covered	Covered	Not covered	Covered	Covered	Not covered	Not covered	Covered	Covered	Covered
	Privacy and protection	Covered	References European Union document	Covered	Covered	Covered	Covered	Covered	Covered	Covered	Covered	Covered	Covered	Covered
	Commitment and collaboration	Covered	Covered	Covered	Covered	Covered	Covered	Covered	Covered	Not covered	Covered	Covered	Covered	Covered
UNESCO	Respect, protection, and promotion of human rights, fundamental freedoms, and human dignity	Compliant	NA	Compliant	Compliant	Compliant	Compliant	Compliant	Compliant	Compliant	Compliant	Compliant	Compliant	Compliant
	Environmental prosperity and ecosystem health	Compliant	NA	Compliant	Compliant	Compliant	Compliant	Non-compliant	Compliant	Non-compliant	Non-compliant	Non-compliant	Compliant	Non-compliant
	Ensuring diversity and inclusion	Compliant	NA	Compliant	Compliant	Compliant	Compliant	Compliant	Compliant	Non-compliant	Non-compliant	Compliant	Compliant	Compliant
	Living in peaceful, just, and interconnected societies	Compliant	NA	Non-compliant	Non-compliant	Non-compliant	Compliant	Compliant	Compliant	Non-compliant	Non-compliant	Non-compliant	Compliant	Compliant
	Proportionality and safety	Compliant	NA	Compliant	Compliant	Compliant	Compliant	Compliant	Compliant	Non-compliant	Compliant	Non-compliant	Compliant	Compliant
	Security and protection	Compliant	NA	Compliant	Compliant	Compliant	Compliant	Compliant	Compliant	Non-compliant	Compliant	Compliant	Compliant	Compliant
	Fairness and non-discrimination	Compliant	NA	Compliant	Compliant	Compliant	Compliant	Compliant	Compliant	Compliant	Compliant	Non-compliant	Compliant	Compliant
	Sustainability	Compliant	NA	Compliant	Compliant	Compliant	Compliant	Compliant	Compliant	Non-compliant	Non-compliant	Compliant	Compliant	Non-compliant
	Right to privacy and data protection	Compliant	NA	Compliant	Compliant	Compliant	Compliant	Compliant	Compliant	Compliant	Compliant	Compliant	Compliant	Compliant
	Human oversight and decision-making	Compliant	NA	Compliant	Compliant	Compliant	Compliant	Compliant	Compliant	Non-compliant	Compliant	Non-compliant	Compliant	Compliant
	Transparency and explainability	Compliant	NA	Compliant	Compliant	Compliant	Compliant	Compliant	Compliant	Non-compliant	Compliant	Compliant	Compliant	Compliant
	Responsibility and accountability	Compliant	NA	Compliant	Compliant	Compliant	Compliant	Compliant	Compliant	Non-compliant	Compliant	Compliant	Compliant	Compliant
	Awareness and education	Compliant	NA	Compliant	Compliant	Non-compliant	Compliant	Compliant	Compliant	Non-compliant	Compliant	Non-compliant	Compliant	Compliant
	Adaptive and multi-stakeholder governance and collaboration	Compliant	NA	Compliant	Compliant	Compliant	Compliant	Compliant	Compliant	Non-compliant	Non-compliant	Compliant	Compliant	Compliant
UN	Inclusiveness	Compliant	NA	Compliant	Compliant	Compliant	Compliant	Compliant	Compliant	Compliant	Non-compliant	Compliant	Compliant	Compliant
	Public interest	Compliant	NA	Compliant	Compliant	Compliant	Compliant	Compliant	Compliant	Compliant	Compliant	Compliant	Compliant	Compliant
	Centrality of data governance	Compliant	NA	Compliant	Compliant	Compliant	Compliant	Compliant	Compliant	Compliant	Compliant	Non-compliant	Compliant	Compliant
	Universal and interconnected governance	Compliant	NA	Compliant	Compliant	Compliant	Compliant	Compliant	Compliant	Non-compliant	Non-compliant	Compliant	Compliant	Compliant
	Governance in accordance with international laws	Compliant	NA	Compliant	Compliant	Compliant	Compliant	Compliant	Compliant	Compliant	Compliant	Compliant	Compliant	Compliant

The analysis of the survey data yields significant insights into the perceptions and attitudes of healthcare professionals toward integrating AI in healthcare. These findings are presented through detailed tables and bar graphs, offering a comprehensive overview of the data.

Table 2 provides a comprehensive overview of the demographic breakdown of survey respondents, including age groups, gender, and professional backgrounds. The data show that most participants fall within the 25-34 and 35-44 age groups, indicating a predominantly young demographic among healthcare professionals. The gender distribution reveals a near-equal balance between female and male participants, ensuring diverse perspectives within the healthcare community. In terms of profession, physicians are the most prominently represented group, followed by pharmacists, nurses, microbiologists, and psychologists. 

**Table 2:** Demographic information of healthcare professionals

Category	Variable	Number of healthcare professionals
Age (years)	Under 25	2
	25-34	25
	35-44	24
	45-54	12
	55 or older	17
Gender	Female	41
	Male	39
Profession	Physician	44
	Pharmacist	13
	Nurse	11
	Microbiologist	10
	Psychologist	2

**Figure 1:**
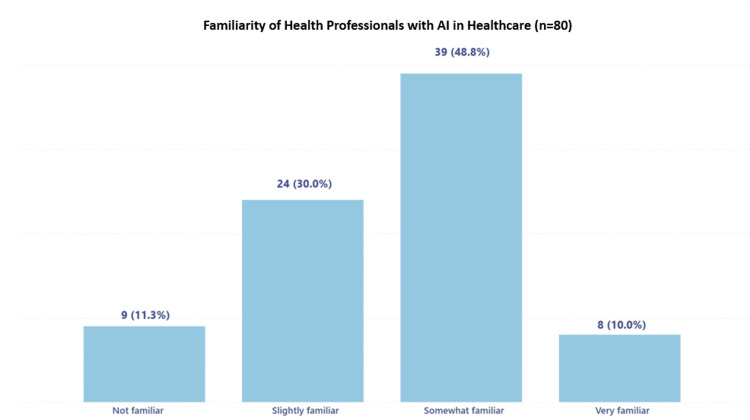
Familiarity of health professionals with AI in healthcare AI: artificial intelligence

Figure 1 depicts the percentage of responses regarding the familiarity of health professionals with AI applications in healthcare, shedding light on the current awareness and knowledge base within the sector.

Many respondents indicated being "Somewhat familiar" with AI, suggesting moderate exposure to AI systems at the hospital where the survey was conducted.

**Figure 2:**
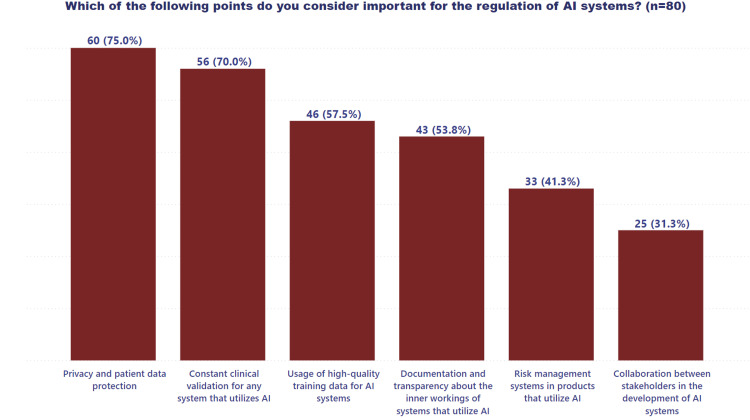
Priorities for the regulation of AI systems in healthcare AI: artificial intelligence

Figure 2 analyzes the priorities identified by various health professionals for the regulation of AI systems in healthcare, offering a nuanced perspective on stakeholder expectations.

Most respondents identified data privacy protection and ongoing clinical validation by healthcare personnel as the highest priority factors in regulating AI systems in healthcare.

**Figure 3:**
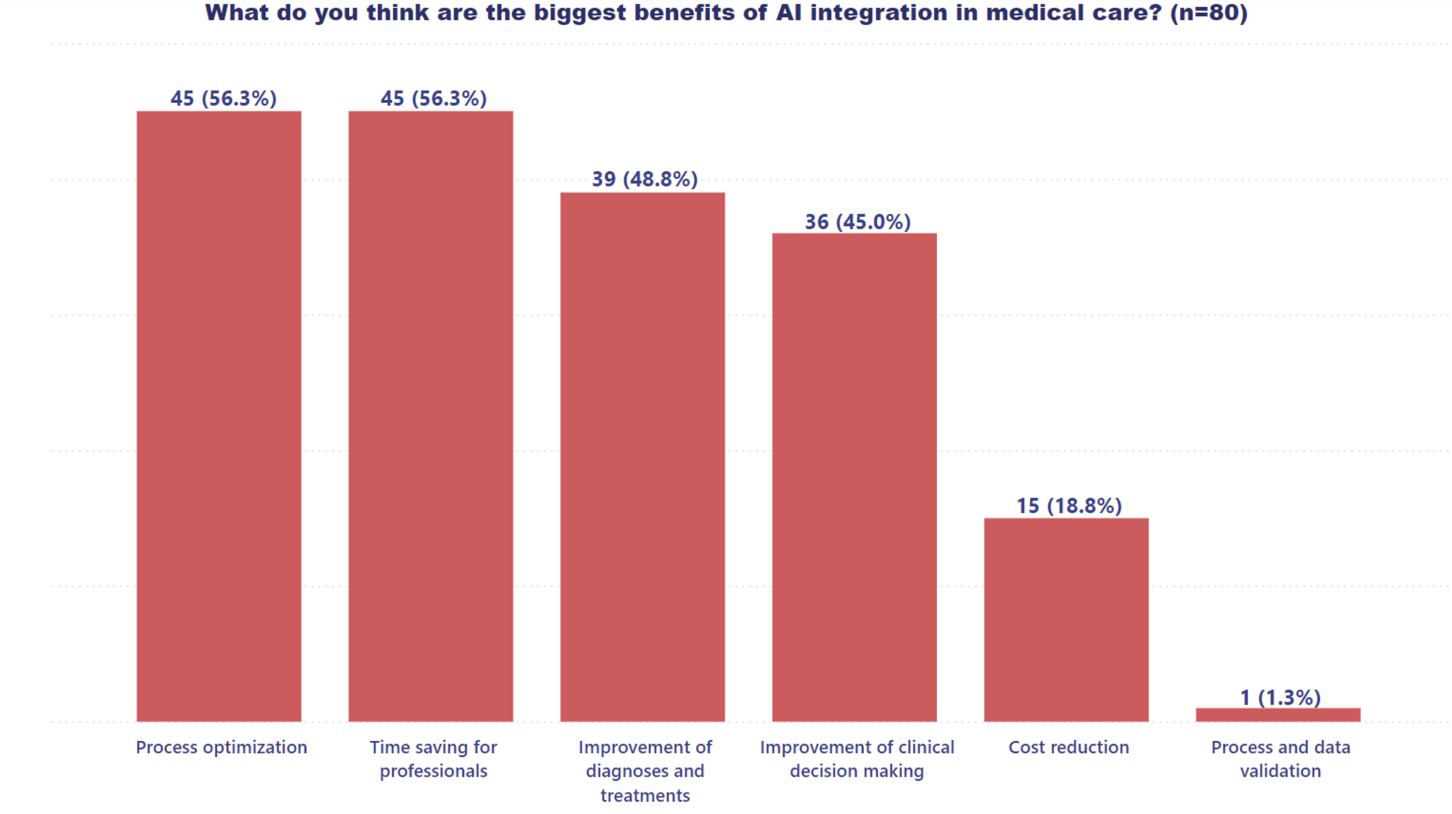
Benefits of integrating AI in healthcare AI: artificial intelligence

Furthermore, Figure 3 highlights the perceived benefits of integrating AI into healthcare, emphasizing the potential advantages and opportunities that these technologies may offer.

Time and process optimization were perceived as the most promising benefits among the options presented, reflecting the direct impact AI could have on healthcare. Additionally, improved diagnosis and treatment and enhanced decision-making were considered key benefits that integrating AI systems could offer the healthcare field.

**Figure 4:**
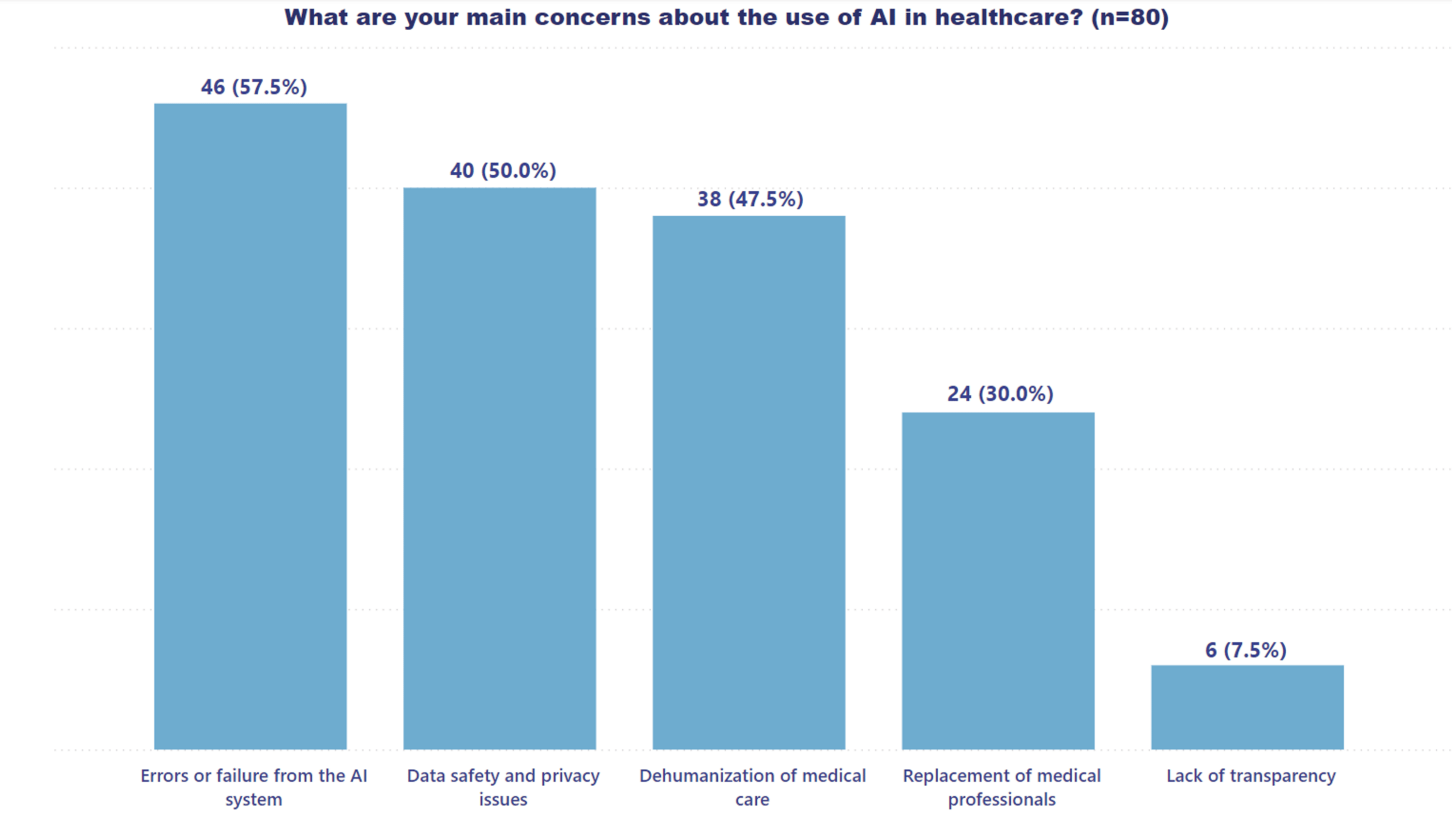
Concerns about using AI systems in healthcare AI: artificial intelligence

Conversely, Figure 4 captures the concerns and apprehensions associated with the use of AI systems in healthcare, providing a balanced understanding of both the promises and challenges.

System failure and data privacy emerged as the top concerns, underlining the critical importance of ensuring the security of sensitive information and the operational stability of AI systems.

**Figure 5:**
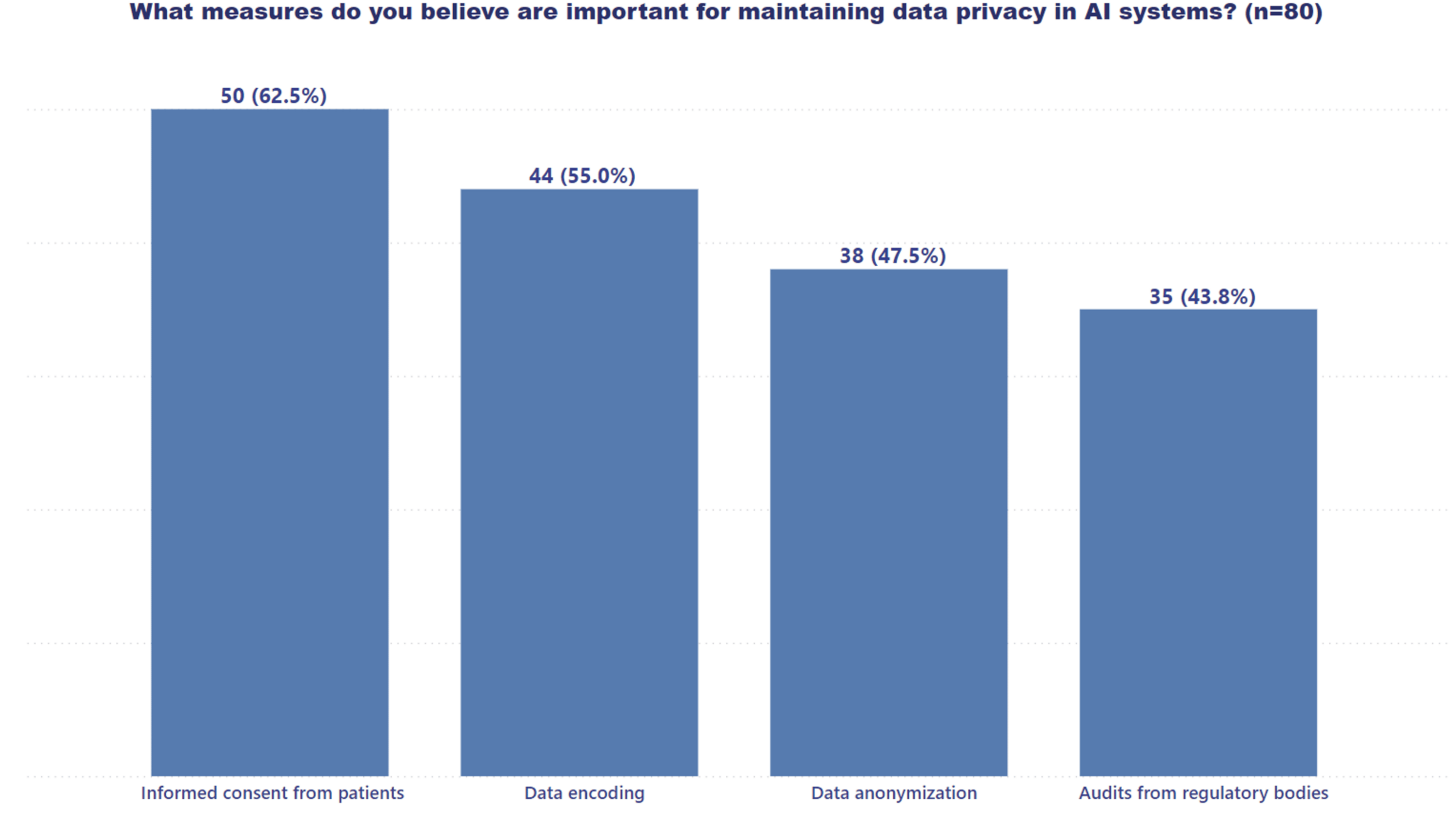
Healthcare professionals' perspectives on key strategies for maintaining AI data privacy AI: artificial intelligence

Figure 5 illustrates healthcare professionals' strong emphasis on obtaining informed consent from patients as a primary strategy for safeguarding AI data privacy. A significant majority (61.7%) of respondents highlighted the importance of securing explicit patient permission before utilizing their health data for AI purposes.

Table 3 presents the statistical analysis of demographic variables, including age, gender, and profession, in relation to key topics concerning AI in healthcare. The p-values obtained from the chi-squared tests indicate that none of these demographic factors showed statistically significant differences in responses, suggesting that familiarity with AI, perceptions of informed consent, and views on ethical regulations were relatively uniform across different groups.

**Table 3:** Statistical analysis of demographic variables and AI-related topics AI: artificial intelligence

Variable	Topic	P-value
Age	AI familiarity	0.441
	Ethical necessity of informed consent	0.119
	Perceptions of the sufficiency of ethical regulations governing AI in healthcare	0.295
Gender	AI familiarity	0.119
	Ethical necessity of informed consent	0.134
	Perceptions of the sufficiency of ethical regulations governing AI in healthcare	0.931
Profession	AI familiarity	0.119
	Ethical necessity of informed consent	0.159
	Perceptions of the sufficiency of ethical regulations governing AI in healthcare	0.482

Regarding AI familiarity, the analysis revealed no significant variation in responses based on age (p=0.441), gender (p=0.119), or profession (p=0.119). This suggests that knowledge and awareness of AI applications in healthcare are not strongly influenced by these demographic factors, indicating a similar level of exposure and understanding across respondents.

In terms of the ethical necessity of informed consent, the results showed no significant differences among respondents when considering whether obtaining informed consent for AI tools in healthcare is an ethical obligation. The p-values for age (p=0.119), gender (p=0.134), and profession (p=0.159) indicate that this ethical concern is widely recognized across different demographic groups, reinforcing the idea that informed consent is a broadly accepted principle in medical practice regardless of professional background or demographic attributes.

Finally, when assessing perceptions of the sufficiency of ethical regulations governing AI in healthcare, no significant differences were found based on age (p=0.295), gender (p=0.931), or profession (p=0.482). This suggests that skepticism or concerns about regulatory frameworks are common across all groups, reflecting a general perception that current ethical guidelines may be insufficient or require further development.

## Discussion

This study focused on assessing healthcare professionals' perceptions at Hospital Clínica Bíblica in Costa Rica regarding the potential and impact of AI in the healthcare field. Most respondents (49%) reported a moderate familiarity with AI, indicating moderate exposure to AI systems within the hospital environment where the study was conducted. While it is common to find healthcare professionals who perceive themselves as advanced technology users, our findings, consistent with previous studies, suggest a trend towards an intermediate self-assessment regarding knowledge and handling of AI tools [27,28]. This result indicates that, even among those with a greater affinity for technology, there is room for improvement in the understanding and application of AI in the clinical setting. 

This study on AI in healthcare provides detailed insights into healthcare professionals' perceptions and attitudes regarding its use and regulation. Both opportunities and challenges in integrating AI into the healthcare sector were identified, highlighting the need for strong governance to address ethical and regulatory concerns. Survey responses revealed several critical areas requiring attention, especially regarding data protection and ongoing clinical validation. 

The demographic profile of participants shows that the majority belong to the age groups between 25 and 44 years, suggesting that younger professionals might be more willing to adopt emerging technologies such as AI. However, the data does not show a clear correlation between familiarity with AI and demographic factors such as age, gender, or profession. This suggests that adopting these systems depends more on the work environment and technical training than on personal characteristics. Studies have determined that these cases may be due to the lack of implementation of AI tools in our healthcare systems [29]. A key finding of the study is the priority that professionals give to data privacy protection and continuous clinical validation in the regulation of AI in healthcare. 

When identifying the highest priority factors for the regulation of AI systems in healthcare, participants mainly highlighted data privacy protection and the need for continuous clinical validation by healthcare personnel. Previous studies have documented the concerns of healthcare professionals regarding implementing these tools [30]. Privacy protection is considered fundamental not only from an ethical point of view but also from a legal and social perspective, given the sensitivity of medical data. Furthermore, continuous clinical validation is seen as essential to ensure that AI systems remain aligned with ethical and clinical standards, avoiding risks that may compromise the quality of care. Privacy protection and the right to data withdrawal must coexist with the need to harness the potential of AI in healthcare [31,32]. 

In a study conducted in Portugal, where healthcare professionals were surveyed, participants indicated that most tasks, especially in hospital settings, could be delegated to AI tools [27].  

Although the physicians surveyed in this study do not express a predominant concern about being replaced by AI, they do recognize the need for specialized training to maximize the potential of this technology. However, they also express concerns about the social impact of AI, such as the possible displacement of other health professionals or the diminution of their professional reputation [33]. 

Significant volumes of confidential information are managed in the healthcare sector, and patients may be reluctant to share this kind of medical information through smart health solutions due to previous incidents of data privacy and security breaches [34,35]. Therefore, the implementation of anonymization mechanisms and the generation of synthetic data can contribute to balancing these concerns. It is also essential to establish a solid regulatory framework that encourages technological innovation while guaranteeing data security. In this way, hospitals will be able to efficiently manage large volumes of information, facilitating the development of increasingly sophisticated diagnostic tools [32,36]. 

Despite the benefits that AI can bring, such as optimizing operational times and processes, concerns have also been raised about data security and privacy. Professionals fear that flaws in AI systems could lead to misdiagnoses or unauthorized exposure of sensitive information [37]. This underlines the need to establish strict regulatory frameworks and appropriate oversight mechanisms to ensure the operational stability and reliability of AI systems, ensuring that they contribute positively to the quality of healthcare without compromising patient safety and privacy [38]. 

The implementation of AI in healthcare poses challenges in both developed and developing countries. While the European Union has taken the lead in regulating and promoting AI in the healthcare sector, Latin American countries and other regions face a significant lag in the adoption of these technologies and the development of adequate regulatory frameworks [27,39]. 

To prevent issues such as discrimination and ensure data security, it is vital to promote transparency in the use of these technologies. Transparency is key to protecting data privacy, as it ensures that AI systems are understandable and explainable and that their limitations and uncertainties are communicated [3]. However, opacity in AI usage remains a problem, as certain processes or algorithms can remain "hidden," compromising transparency. In some cases, developers protect the functioning of their AI systems as trade secrets; in others, opacity arises because the complex programming techniques and algorithms that underpin these technologies are difficult for the general public to understand [36]. 

Diverse national policies and attitudes towards AI globally have led to the uneven implementation of this technology, limiting its potential to improve patient care and protect personal data. Countries must work in a coordinated manner at regional and global levels to overcome these barriers and ensure equitable and ethical implementation of AI in the healthcare sector. International cooperation is essential to share knowledge, establish common standards, and promote the development of innovative solutions that benefit all citizens. 

This study's scope, limited to a single private hospital, restricts the generalizability of its results to other institutional contexts. The specific characteristics of this center, including resources, internal organization, and the population served, can significantly influence professional perceptions and practices. Future research should include a more diverse sample of institutions to identify both unique and common elements across the healthcare sector. 

The absence of consistent regulatory frameworks for AI in Latin America poses challenges for policy implementation and cross-country comparisons. As a qualitative and descriptive cross-sectional study, it captures perceptions and attitudes at a specific point in time, preventing the observation of changes or trends. The sample, composed solely of health professionals from Hospital Clínica Bíblica in Costa Rica, may limit generalizability to other contexts, such as public hospitals or different regions. Voluntary participation may introduce selection bias, as those more interested or knowledgeable about AI might be more inclined to participate. 

## Conclusions

This study highlights the promising yet complex integration of AI in healthcare, with Costa Rican professionals recognizing its potential to enhance diagnostics, treatments, and personalized care. However, addressing concerns about data privacy, clinical validation, ethics, and the need for training and regulation is crucial. These findings underscore the importance of interdisciplinary collaboration and longitudinal studies to ensure AI's successful implementation and positive impact on healthcare.

## Appendices

Data collection tool

Perspective of Healthcare Professionals on the Integration of Artificial Intelligence

1. Age

(a) Under 25 years

(b) 25-34

(c) 35-44

(d) 45-54

(e) 55 or older

2. Gender

(a) Female

(b) Male

3. Profession

(a) Physician

(b) Pharmacist

(c) Nurse

(d) Microbiologist

4. Years of experience

(a) Less than 5 years

(b) Between 5 and 15 years

(c) More than 15 years

5. Department

(a) Pharmacy

(b) ICU

(c) Emergency

(d) Internal Medicine

(e) Nuclear Medicine

(f) Radiology

(g) Laboratory

(h) Others

6. Are you familiar with the applications of artificial intelligence in the healthcare sector?

(a) Very familiar

(b) Somewhat familiar

(c) Slightly familiar

(d) Not familiar

7. Greatest benefit of artificial intelligence

(a) Improvement in diagnosis and treatment

(b) Time saving

(c) Cost reduction

(d) Improvement in decision-making

(e) Process optimization

(f) Others

8. What are your main concerns about the use of artificial intelligence in healthcare?

(a) Privacy and security

(b) Dehumanization

(c) Lack of transparency

(d) Error or system failure

(e) Replacement of professionals

(f) Others

9. Do you believe there are sufficient ethical regulations to govern the use of artificial intelligence in healthcare?

(a) Yes

(b) No

(c) Not sure

10. Are you aware if there are ethical regulations governing the use of artificial intelligence in Costa Rica?

(a) Yes

(b) No

(c) Not sure

11. How concerned are you about patient data privacy when using artificial intelligence systems?

(a) Very concerned

(b) Somewhat concerned

(c) Slightly concerned

(d) Not concerned

12. What measures do you think are most important to protect data privacy in artificial intelligence systems?

(a) Data encryption

(b) Data anonymization

(c) Informed consent

(d) Regulatory audits

(e) Others

13. Are you aware of artificial intelligence and ethics regulations developed by organizations such as the World Health Organization (WHO), the United Nations Educational, Scientific and Cultural Organization (UNESCO), and the United Nations (UN)?

(a) Yes

(b) No

(c) Not sure

14. Which of the following points do you consider most important for the regulation of artificial intelligence systems?

(a) Documentation and transparency

(b) Risk management systems

(c) Continuous clinical validation

(d) Use of high-quality data

(e) Privacy and data protection

(f) Stakeholder collaboration

(g) Others

15. Do you consider providing informed consent to your patient about the use of artificial intelligence tools an ethical necessity?

(a) Yes, absolutely necessary

(b) Depends on the situation

(c) No, not necessary

(d) Not sure

16. Informing about artificial intelligence use

(a) Yes, always

(b) Only if I consider it relevant

(c) No, unless the patient asks

(d) Not sure

17. Comfort explaining artificial intelligence to patients

(a) Very comfortable

(b) Somewhat comfortable

(c) Slightly comfortable

(d) Not comfortable

18. Impact of artificial intelligence on doctor-patient relationship

(a) Would improve the relationship

(b) Would make it more impersonal

(c) Depends on the use and communication

(d) Not sure

19. Importance of transparency

(a) Very important

(b) Somewhat important

(c) Slightly important

(d) Not important

20. Concern about lack of transparency

(a) Yes, very concerning

(b) Somewhat concerning

(c) Not very concerning

(d) Don't think it's a problem

21. Responsibility in case of error

(a) Mainly on the healthcare professional

(b) Mainly on the artificial intelligence system

(c) Depends on the situation

(d) Not sure
